# Rapid Identification of Functional Pyrrolysyl-tRNA Synthetases via Fluorescence-Activated Cell Sorting

**DOI:** 10.3390/ijms20010029

**Published:** 2018-12-21

**Authors:** Andrew E. Lin, Qing Lin

**Affiliations:** Department of Chemistry, State University of New York at Buffalo, Buffalo, NY 14260-3000, USA; alin25@buffalo.edu

**Keywords:** amber codon suppression, genetic code expansion, flow cytometry, mutagenesis, non-natural amino acids

## Abstract

The orthogonal pyrrolysyl-tRNA synthetase/tRNA_CUA_ pair and their variants have provided powerful tools for expanding the genetic code to allow for engineering of proteins with augmented structure and function not present in Nature. To expedite the discovery of novel pyrrolysyl-tRNA synthetase (PylRS) variants that can charge non-natural amino acids into proteins site-specifically, herein we report a streamlined protocol for rapid construction of the pyrrolysyl-tRNA synthetase library, selection of the functional PylRS mutants using fluorescence-activated cell sorting, and subsequent validation of the selected PylRS mutants through direct expression of the fluorescent protein reporter using a single bacterial strain. We expect that this protocol should be generally applicable to rapid identification of the functional PylRS mutants for charging a wide range of non-natural amino acids into proteins.

## 1. Introduction

The discovery of the pyrrolysyl-tRNA synthetase (PylRS) and its cognate tRNA, tRNA^Pyl^, from *Methanosarcina barkeri*, which are capable of charging the 22nd natural amino acid, pyrrolysine, into proteins in response to an amber codon in methanogens, has unleashed a new era of protein engineering [1,2]. Since this discovery, the PylRS and its many variants have been obtained through design and/or evolution to charge a large number of non-natural amino acids, including more than 100 pyrrolysine and phenylalanine analogs, site-specifically into proteins in *Escherichia coli* as well as in mammalian cells for production of engineered proteins with enhanced properties [3,4]. Despite this rapid progress, a major bottleneck of the field is the lack of general methods for *rapid* identification of the functional PylRS variants for charging any designed non-natural amino acids. Current methods principally rely on either the survival-based selections [5] or the phage-assisted evolution in bacteria [6], both of which are time-consuming and involve complicated procedures.

Fluorescence-activated cell sorting (FACS) has been used for the selection and screening functional aminoacyl-tRNA synthetases from the designed libraries. For example, Schultz and co-workers reported the use of green fluorescent protein (GFP) as a fluorescent reporter, whose expression is regulated by an amber-codon-containing T7 RNA polymerase [7]. Because of the high background due to the non-natural amino acid-independent expression of GFP, three rounds of positive selections and one round of negative selection were performed successively in order to identify the functional aminoacyl-tRNA synthetases. Similarly, Skerra and co-workers successfully carried out alternating rounds of positive and negative fluorescence-activated cell sorting of *E. coli* cells in the presence and absence, respectively, of the non-natural amino acid, *O*-methyl-L-tyrosine, using a one-plasmid expression system encoding inducible modified *Methanocaldococcus jannaschii* (*Mj*) tyrosyl-tRNA synthetase, orthogonal cognate suppressor tRNA, and an enhanced GFPamber mutant [8]. The drawback of including negative selection is that the activity and specificity of the isolated synthetase variants are restricted by the dynamic range of the negative selection because the most active clones are often deleted from the library pool during the negative selection [9]. On the other hand, Tirrell and co-workers developed a FACS-based screening protocol to examine the capability of a library of methionyl-tRNA synthetase variants in residue-specific incorporation of an azide-containing non-natural amino acid into proteins [10]. To enable fluorescence-based FACS sorting, an amber codon was inserted into the coding region of the *E. coli* outer membrane protein OmpC such that the genetically encoded azide group can be selectively functionalized with a fluorescent tag via click chemistry on bacterial surface. The limitation of this method is that it is only suitable for screening the aminoacyl-tRNA synthetase variants for reactive non-natural amino acids, as the fluorophore attachment requires a reactive ‘war-head’ that is not present in most non-natural amino acids.

**Scheme 1:**
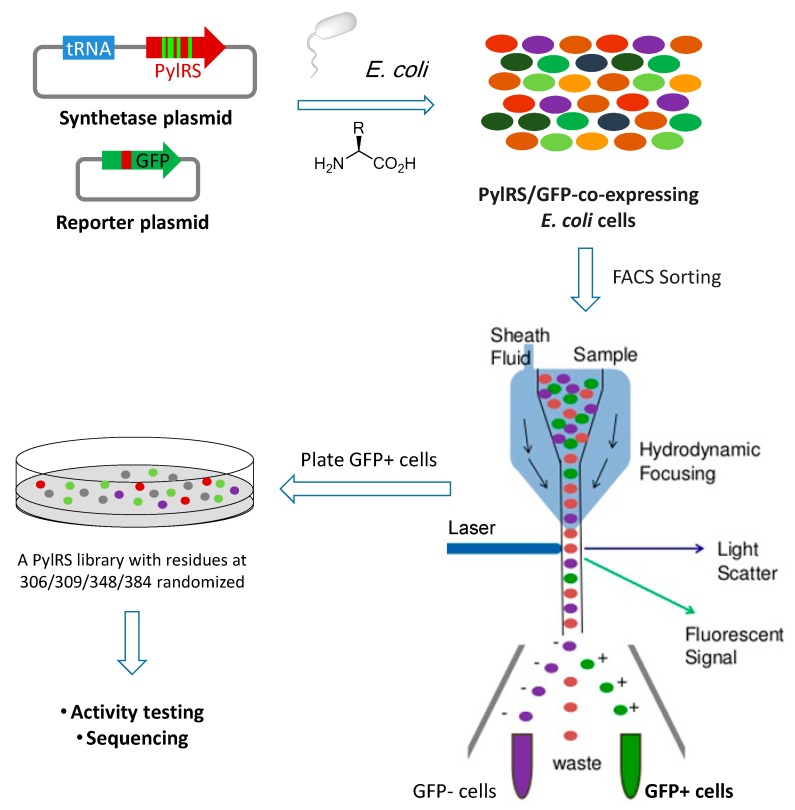
Fluorescence-activated cell sorting (FACS)-based selection of functional pyrrolysyl-tRNA synthetase (PylRS) variants from a synthetase library. Two plasmids, one encoding PylRS/tRNA^Pyl^ and the other encoding the green fluorescent protein (GFP)-204TAG reporter, are co-transformed into *Escherichia coli* cells, and fluorescent *E. coli* cells are generated through induction of PylRS and GFP-204TAG expression in the presence of a non-natural amino acid. The color variation in oval-shaped *E. coli* cells illustrates variation of GFP expression levels. The resulting *E. coli* cells are sorted based on GFP fluorescence. The GFP+ *E. coli* cells are collected and plated, and the selected clones are subjected to activity testing and DNA sequencing.

In this work, we report a facile screening protocol based on fluorescence-activated cell sorting (FACS) in *E. coli*, which allowed us to identify a functional PylRS variant from the designed PylRS library within a week. This selection procedure is unbiased, as it directly assesses the full range of the suppression activities of the encoded PylRS variants en mass in the entire library in supporting the expression of the non-natural amino-acid-encoded fluorescent reporter protein carrying an amber codon in its coding region (Scheme 1).

## 2. Results

### 2.1. Construction of the PylRS Library

**Figure 1:**
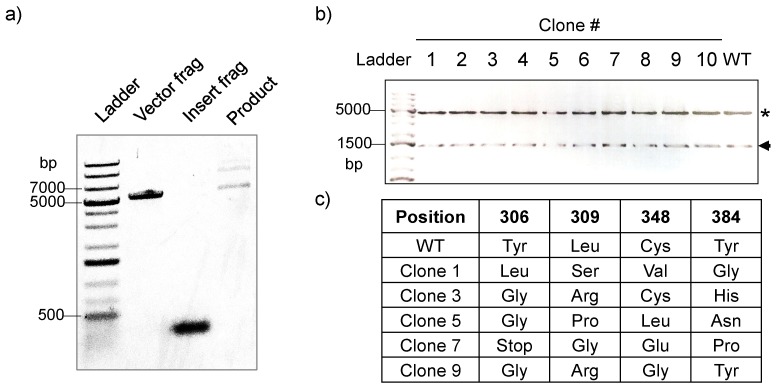
Construction of the PylRS library using Gibson assembly. (**a**) Agarose gel electrophoresis of the DNA fragments and the assembled product mixture. (**b**) Agarose gel electrophoresis of 10 clones randomly picked from a Luria–Bertani (LB)-agar plate containing 34 µg/mL chloramphenicol. The plasmids were digested with the restriction enzymes *Sal*I and *Spe*I at 37 °C for 2 h prior to electrophoresis. The asterisk indicates the vector fragment, while the arrow indicates the PylRS insert. (**c**) DNA sequencing results of five PylRS plasmids, showing the amino acid identities at the randomized positions.

Since a large fraction of the functional PylRS variants contain mutations at the following four residues in their amino acid binding pockets [4]: Tyr306, Leu309, Cys348, and Tyr384, we constructed a relatively small PylRS library by randomizing these four residues with a calculated library size of 1.6 × 10^5^. Specifically, we employed the exonuclease-based Gibson assembly method because of its seamless assembly of DNA fragments in a pre-defined order [11]. In brief, the PylRS DNA insert containing the randomized regions was obtained through overlapping PCR of the two appropriate fragments: one contains randomization at residues 306 and 309, and the other contains randomization at residues 348 and 384. The full-length PylRS library was assembled by mixing the vector fragment with the insert in a 1:3 molar ratio in an Eppendorf tube containing an equal volume of 2× HiFi DNA Assembly master mix at 50 °C for 1 h. Analysis of the product by agarose gel electrophoresis showed successful assembly of the fragments into the PylRS plasmid library (Figure 1a). To assess library quality, the plasmids from 10 randomly selected clones were digested with *Sal*I/*Spe*I and analyzed by agarose gel electrophoresis. The gel showed the presence of the two fragments identical to those from the wild-type, indicating a high degree of assembly efficiency (Figure 1b). Sequencing of the PylRS plasmids from five selected clones revealed no clear preference at any of the amino acid positions (Figure 1c), suggesting that there is no intrinsic bias in the fragment assembly.

### 2.2. Assessment of PylRS Library Activity via FACS Analysis

**Figure 2:**
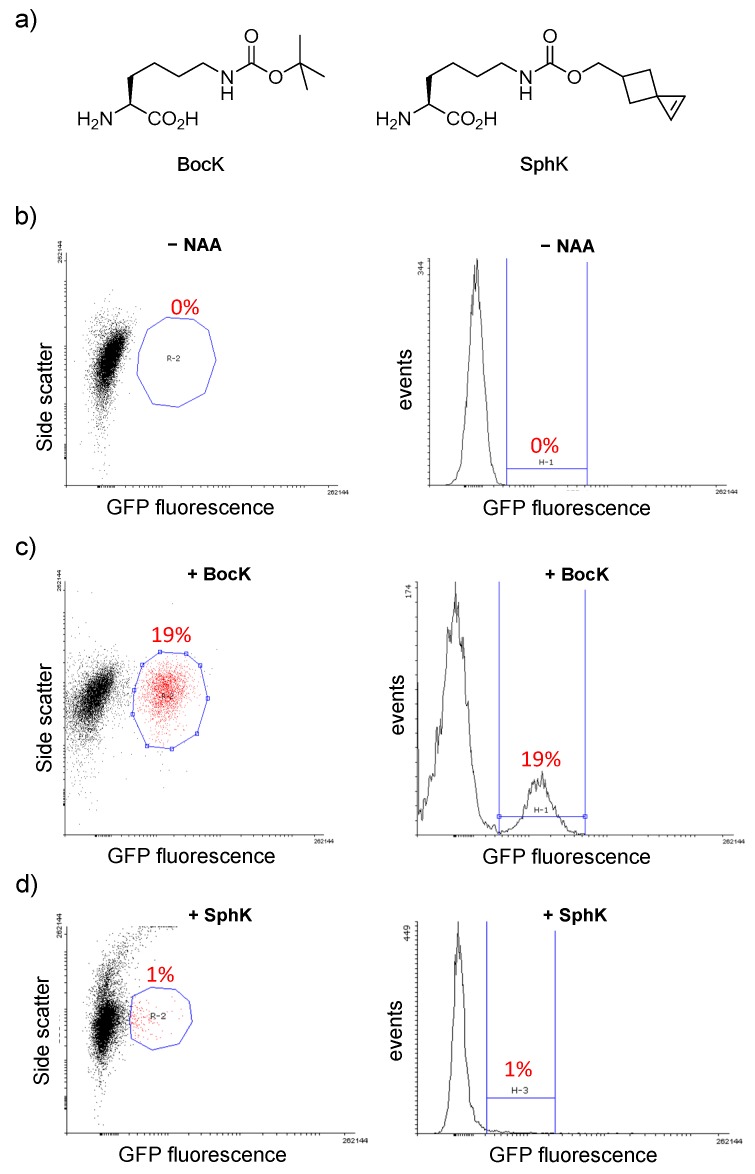
FACS analyses of the PylRS library activity. (**a**) Structures of BocK and SphK. (**b**–**d**) FACS analysis of Acella cells co-expressing the PylRS library and sfGFP-204TAG in the absence of any non-natural amino acid (**b**) or the presence of 2 mM BocK (**c**) or the presence of 1 mM SphK (**d**). The regions showing GFP+ cells are circuled and quantified in the dot blots. The percentage of fluorescent cells is also marked in the hostograms. Ten thousand events were recorded for each FACS analysis.

To enable both plasmid amplification and protein expression using a single bacterial strain, we prepared electrocompetent Acella™ cells, a derivative of BL21(DE3), with additional deletions of the *endA* and *recA* genes, pretransformed with the pET-sfGFP-204TAG reporter plasmid encoding an amber codon at position-204. The PylRS library was transformed into Acella cells, and the transformants were selected on an ampicillin and chloramphenicol-containing Luria–Bertani (LB)-agar plate. The surviving colonies were collected into 10-mL LB medium, and the expressions of sfGFP-204TAG and the PylRS variant were induced with the addition of appropriate amounts of IPTG and arabinose, respectively, in the presence or absence of a non-natural amino acid. The cells were pelleted, washed with phosphate-buffered saline (PBS), and resuspended in 10% glycerol for FACS analysis. As expected, cells grown in the absence of any non-natural amino acids did not produce fluorescent cells in the GFP+ region (Figure 2b), indicating that the background amber suppression with endogenous amino acids by the PylRS library is negligible. In the presence of BocK (structure shown in Figure 2a), a non-natural amino acid used previously in studies of the PylRS substrate specificity, 19% of cells showed green fluorescence, indicating that a large fraction of the PylRS library is capable of charging BocK into the sfGFP-204TAG mutant protein (Figure 2c). This result is consistent with the earlier reports that both the wild-type and mutant PylRS enzymes can recognize BocK as a substrate [12,13,14]. To probe the generality of the FACS screening, we also induced the reporter protein expression in the presence of SphK (structure shown in Figure 2a), a non-natural amino acid that can be charged by the wild-type PylRS [15], and performed the same FACS screening. Interestingly, a significantly smaller fraction of Acella cells (~1%) exhibited green fluorescence (Figure 2d), indicating that SphK is a much more restrictive substrate than BocK for the PylRS library.

### 2.3. Identification of Functional PylRS Variants via FACS Sorting and Verification of Their Activity

**Figure 3:**
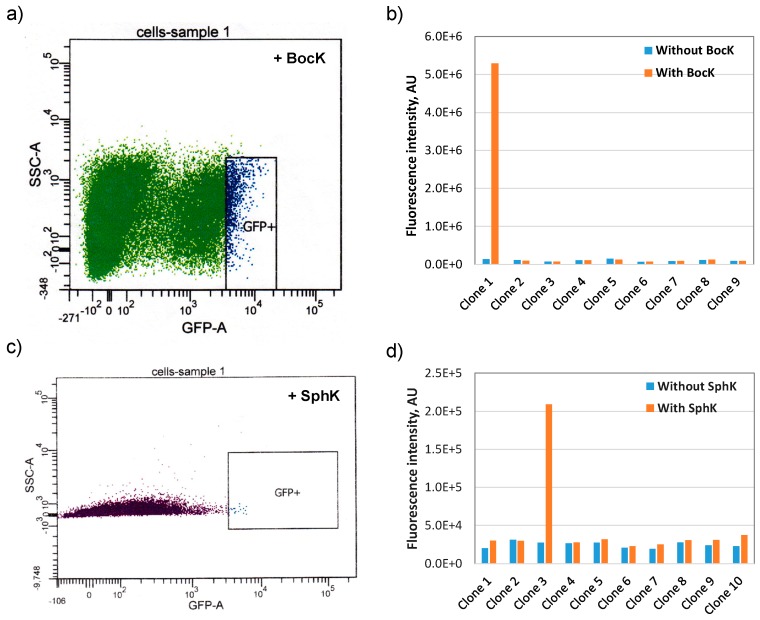
Identification of the active PylRS variants via FACS sorting. (**a**,**c**) Dot plots showing the sorting of Acella cells co-expressing the PylRS library and the fluorescent sfGFP-204TAG reporter in the presnce of (**a**) BocK or (**c**) SphK. The GFP+ cells rendered in blue were collected. SSC-A represents side scatter. (**b**,**d**) Fluorescence intensity of the cell lysates from the outgrowth of the randomly picked individual clones in LB medium after induction of protein expression in the presence of 1 mM of (**b**) BocK or (**d**) SphK. The GFP fluorescence was measured using a microtiter fluorescence plate reader with *λ*_ex_ set at 485 nm and *λ*_em_ set at 510 nm.

Having established that Acella cells are suitable for both recombinant expression of a fluorescent reporter protein encoding a non-natural amino acid and subsequent fluorescence-based screening, we proceeded to isolate the active PylRS clones through FACS sorting and determine their identities through DNA sequencing. Accordingly, two tubes of Acella cells co-expressing the PylRS library and sfGFP-204TAG in the presence of 1 mM BocK or SphK were sorted using a BD FACSAria II cell sorter. For the BocK-encoded sample, 5000 GFP+ cells were collected over ~8 min after sorting 5.6 million cells at a sort rate of ~7 × 10^5^ event/min (Figure 3a). The collected cells were further selected on an LB-agar plate containing appropriate amounts of ampicillin and chloramphenicol. Nine colonies were then randomly picked from the plate and tested for their ability to incorporate BocK into sfGFP-204TAG in liquid culture. Among them, one clone showed excellent activity, with the fluorescence intensity increased by roughly 39-fold over the background (Figure 3b). Sequencing of this clone revealed that the randomized residues match those of the wild-type (Y306, L309, C348, and Y384). This result is not surprising, given that the wild-type PylRS enzyme has shown robust activity in charging BocK [12]. Similarly, for the SphK-encoded sample, 500 GFP+ cells were collected over ~1 min from roughly 2.2 million Acella cells at a sort rate of ~3 × 10^6^ event/min (Figure 3c). After plating cells onto an LB-agar plate containing appropriate amounts of ampicillin and chloramphenicol, 10 surviving colonies were randomly picked and examined for their ability to charge SphK into sfGFP-204TAG in liquid culture. Again, only one clone showed SphK-dependent expression of the sfGFP-204TAG mutant, with the fluorescence intensity increased by roughly 8-fold over the background (Figure 3d). Sequencing of the corresponding plasmid revealed that the wild-type PylRS was encoded, presumably due to the higher activity associated with the wild-type enzyme [15]. Indeed, more than 22 non-natural amino acids have been reported to be accepted by the wild-type PylRS, a testament to the versatility and promiscuity of the wild-type enzyme as a genetic code expansion tool [4,16,17,18]. Large-scale expressions of the sfGFP-204TAG mutant in Acella cells in the presence of 1 mM BocK or SphK gave the corresponding mutant protein at a yield of 20 and 5.4 mg/L, respectively, similar to what has been reported previously using BL21(DE3) cells as the expression host [15,19].

## 3. Discussion

The screening strategy described here is based exclusively on bacterial sorting, utilizing the fluorescent reporter protein sfGFP equipped with an amber codon in its coding region. Together with the use of a dual-purpose Acella strain, the library generation, screening, and clone amplification and sequencing is extremely facile. With a suitably designed PylRS library in hand, the entire screen and verification can be completed within a week. Compared to the survival-based selection methods, the FACS-based screening method has several advantages. First, FACS is extremely sensitive in detecting low cellular fluorescence, which is particularly important during the screening of novel non-natural amino acids with modest substrate properties. For instance, in the FACS screening of SphK-specific PylRS variants, only ~1% of cells displayed modest sfGFP expression, with the mean fluorescence only 5-fold higher than the library mean (Figure 2d). Second, unlike the plate-based genetic screens in which stringency is controlled by antibiotic concentrations, the FACS-based screen allows for precise in-flight tuning of the selection parameters via appropriate placement of sort gates depending on the percentage of GFP+ clones in the library (Figure 3a,c), leading to a high degree of flexibility. Third, the FACS-based sorting is extremely fast, and up to 10^5^ bacterial cells can be sorted per second using conventional sorters. For instance, we completed our FACS sorting of 6 million cells in 8 min. However, it is usually not practical to use FACS to screen large libraries with a size greater than 1 × 10^8^. One potential solution is to optimize the larger PylRS libraries through customized codon randomization based on the principle of “small-intelligent” mutagenesis, which dramatically reduces the theoretic size of the library without loss of diversity at the critical amino acid positions [20]. Fourth, owing to the small culture volume (usually 1–5 mL) used in the screen, re-amplification, and confirmation, only mg quantities of non-natural amino acids are needed, which is particularly valuable for non-natural amino acids that require many steps to synthesize with low overall yields; e.g., it takes more than 10 steps to prepare SphK [15]. Finally, it should be possible to use other fluorescent proteins, such as mCherry, as a reporter of PylRS activity to further increase signal-to-noise ratio in FACS-based screen due to lower cellular autofluorescence in the red region.

## 4. Materials and Methods

### 4.1. Construction of the PylRS Library Using Gibson Assembly

Fragment 1, containing randomization at residues 306 and 309, was obtained by PCR using pEvol-*mm*PylRS as the template and the following pair of oligos as the primers: 5′-CCATGCTTGCTCCAAACCTTNNKAACTACNNKCGCAAG-3′ and 5′-CCATCTGGCAGAAGTTCAGCATGGTAAAC-3′. Fragment 2, with randomization at residues 348 and 384, was obtained by PCR using pEvol-*mm*PylRS as the template and the following pair of oligos: 5′-GCTGAACTTCNNKCAGATGGGATCGGGA-3′ and 5′-ATTAC ATCAGGGTATCCCCMNNGACCATGCAGGAATCGC-3′. In both reactions, Q5 hot start high-fidelity 2× master mix (New England Biolabs, Ipswich, MA, USA) was used. The gel-purified fragments 1 and 2 were assembled into a single insert by PCR using Phusion high-fidelity DNA polymerase (New England Biolabs, Ipswich, MA, USA) and the following pair of oligos: 5′-CCATGCTTGCTCCAAACCTTNNKAACTACNNKCGCAAG-3′ and 5′-ATTACATCAAGGGTATCCCCMNNGACCATGCAGGAATCGC-3′. The vector fragment was obtained by PCR using pEvol-*mm*PylRS as the template and the following pair of oligos as the primers: 5′-GGGGATACCCTTGATGTAATG-3′ and 5′-AAGGTTTGGAGCAAGCATG-3′. The Gibson assembly was carried out by mixing the vector fragment (5.5 kb, 22 ng, 0.005 pmol) and the insert (250 bp, 4 ng, 0.025 pmol) with 5 µL of 2× HiFi DNA Assembly master mix in a 10 µL total reaction at 50 °C for 1 h. Then, two separate 1-µL of assembly mixtures were transformed into two separate tubes of 50-µL electrocompetent NEB5α cells (New England Biolabs, Ipswich, MA, USA), and the transformants were recovered in SOB medium at 37 °C for 1 h and combined before plating onto five LB-agar plates containing 34 µg/mL chloramphenicol. After overnight incubation in a 37 °C oven, the surviving colonies were collected from the plates and allowed to grow in LB medium containing 34 µg/mL chloramphenicol at 37 °C for 5 h. The PylRS plasmid library was purified using a plasmid mini-prep kit. The concentration of the library plasmid was determined to be 194 ng/µL using Nanodrop 2000c spectroscopy (Thermo Fisher Scientific, Waltham, MA, USA).

### 4.2. Assessment of the PylRS Library Quality

An aliquot of 1-µL Gibson assembly mixture was transformed into NEB-5α electrocompetent cells (New England Biolabs, Ipswich, MA, USA). The cells were allowed to recover in 1 mL of Super Optimal Broth (SOB) medium in a 37 °C incubator–shaker for 1 h. Fifteen microlitres of the mixture were plated onto an LB-agar plate containing 34 µg/mL chloramphenicol. After incubation at 37 °C overnight, 10 colonies were randomly picked from the plate and allowed to grow in 2 mL LB medium containing 34 µg/mL chloramphenicol overnight. The plasmids were purified through mini-prep, digested with *Sal*I/*Spe*I, and analyzed by 1% agarose gel electrophoresis. Five plasmids were submitted for DNA sequencing.

### 4.3. FACS Analysis of Acella^TM^ Cells Co-Expressing the PylRS Library and sfGFP

An aliquot of 1 µL plasmid library (194 ng/µL) was transformed into electrocompetent Acella^TM^ cells (EdgeBio, Gaithersburg, MD, USA; competency = 2 × 10^8^ cfu/µg) carrying the pET-sfGFP-204TAG plasmid, and the transformants were recovered in SOB medium in a 37 °C incubator–shaker for 1 h. Then, 15 µL of the transformation mixture were plated onto an LB-agar plate containing 34 µg/mL chloramphenicol and 50 µg/mL ampicillin. After incubation at 37 °C overnight, the surviving clones were collected into 10 mL LB medium containing 34 µg/mL chloramphenicol and 50 µg/mL ampicillin, and the culture was allowed to grow until the OD_600_ reached ~0.7. Two microlitres of culture were added separately to three 15-mL tubes. In tube 1, appropriate amounts of isopropyl β-d-1-thiogalactopyranoside (IPTG) and arabinose were added to obtain final concentrations of 1 mM and 0.2%, respectively. In tube 2, cells were grown in the presence of 1 mM IPTG, 0.2% arabinose, and 2 mM BocK. In tube 3, cells were grown in LB medium supplemented with 1 mM IPTG, 0.2% arabinose, and 1 mM SphK. The tubes were incubated in an incubator–shaker at 37 °C for 3 h, and then the cells were pelleted in 1.5-mL Eppendorf tubes through centrifugation. The cells were washed successively with PBS (1 mL × 1) and cold 10% glycerol (1 mL × 2), resuspended in 1 mL 10% glycerol, and stored in a −80 °C freezer. For FACS analysis, the samples were loaded into a BD Biosciences LSR Fortessa X-20 flow cytometer and analyzed based on GFP fluorescence. The data were plotted using Flowing 2.5.1 software (University at Turku, Finland).

### 4.4. FACS Sorting of Acella™ Cells Co-Expressing the PylRS Library and sfGFP

The Acella cells in 10% glycerol were thawed at room temperature and then diluted with PBS to an appropriate density before being injected into a BD FACSAriaII cell sorter (BD Biosciences, San Jose, CA, USA) specifically tuned for bacterial sorting. For Acella cells treated with BocK about 5000 GFP+ cells were collected into a 1.5-mL Eppendorf tube containing 200 µL SOB medium from 5.6 million cells. For the SphK-treated cells, roughly 500 cells were collected from 2.2 million cells.

### 4.5. Verification of Biological Activity of the Selected PylRS Variants

The collected Acella cells in 200 µL SOB medium were recovered in a 37 °C incubator–shaker for 1 h before being plated onto an LB-agar plate containing 34 µg/mL chloramphenicol and 50 µg/mL ampicillin. After incubation in a 37 °C oven overnight, 9–10 colonies were randomly picked from the plate and allowed to grow in 2 mL LB medium supplemented with 34 µg/mL chloramphenicol and 50 µg/mL ampicillin at 37 °C. After the OD_600_ reached 0.6–0.8, appropriate amounts of IPTG and arabinose were added to induce protein expression in the absence or presence of 1 mM BocK and SphK, respectively. After 5-h growth at 37 °C, the cells were collected via centrifugation and the pellets were lysed with BugBuster Protein Extraction Reagent (EMD Millipore, Burlington, MA, USA) following the manufacturer’s recommended procedure. The supernatants were transferred to a 96-well plate, and the GFP fluorescence was measured using a Synergy H1 plate reader (BioTek Instruments, Winooski, VT, USA). For the positive clones that produced strong fluorescence, the plasmid mini-preps were performed with the cell pellets and the purified plasmids were submitted for DNA sequencing.

## 5. Conclusions

In summary, we have developed a FACS-based screening protocol for rapid identification of functional pyrrolysyl-tRNA synthetases in bacteria. The notable steps include the efficient construction of the PylRS library using the Gibson assembly method, the use of dual-purpose Acella cells for expression of the PylRS library and subsequent plasmid amplification, and the use of a superfolder GFP mutant carrying an amber codon as a fluorescent reporter of PylRS activity in FACS analysis and sorting as well as functional verification. Using this new protocol, we successfully identified the functional PylRS that can charge both BocK and SphK into proteins site-specifically from a small PylRS library. Having established this proof-of-concept study, we are setting out to employ this protocol to search for PylRS variants that can charge many interesting non-natural amino acids, e.g., the spirohexene-lysine analogs suitable for bio-orthogonal protein labeling of the class B G protein-coupled receptors (GPCRs) in live mammalian cells [21,22].

## Abbreviations

BocK	*N*^ε^-*tert*-butoxycarbonyl-L-lysine
*E. coli*	*Escherichia coli*
FACS	fluorescence-activated cell sorting
GFP	green fluorescent protein
IPTG	isopropyl β-d-1-thiogalactopyranoside
LB medium	Luria–Bertani medium
PBS	phosphate buffered saline
PylRS	pyrrolysyl-tRNA synthetase
sfGFP	super folder green fluorescent protein
SphK	*N*^ε^-spiro[2.3]hex-1-enylmethoxycarbonyl-L-lysine
